# The Zfx gene is expressed in human gliomas and is important in the proliferation and apoptosis of the human malignant glioma cell line U251

**DOI:** 10.1186/1756-9966-30-114

**Published:** 2011-12-20

**Authors:** Youxin Zhou, Zuopeng Su, Yulun Huang, Ting Sun, Sansong Chen, Tingfeng Wu, Guilin Chen, Xueshun Xie, Bin Li, Ziwei Du

**Affiliations:** 1 Neurosurgery & Brain and Nerve Research Laboratory, The First Affiliated Hospital of Soochow University, suzhou 215006,China

**Keywords:** Zfx, U251, Proliferation, Apoptosis

## Abstract

**Background:**

Zfx is a zinc finger protein of the Zfy family, whose members are highly conserved in vertebrates. Zfx is a shared transcriptional regulator of both embryonic stem cells (ESC) and hematopoietic stem cells (HSC), which suggests a common genetic basis of self-renewal in embryonic and adult stem cells. The level of Zfx expression correlates with aggressiveness and severity in many cancer types, including prostate cancer, breast cancer, and leukemia. However, the importance of Zfx in human glioma is largely unknown. In the present study, we examined the role of Zfx in human glioma.

**Methods:**

We detected expression levels of Zfx mRNA in U251 cells, U87 cells, U373 cells, and A172 cells by semi-quantitative RT-PCR. To analyze the expression of Zfx mRNA in glioma tissues, we performed real-time quantitative PCR on 35 pathologically confirmed glioma samples (Grade I-4cases, Grade II-13cases, Grade III-11cases, and Grade IV-7cases) and on 5 noncancerous brain tissue samples. We used lentivirus-mediated small interfering RNAs (siRNAs) to knock down Zfx expression in the human malignant glioma cell line U251. Changes in Zfx target gene expression were determined by real-time RT-PCR. Cell proliferation was examined by a High Content Screening assay. DNA synthesis in proliferating cells was determined by BrdU incorporation. Cell cycle distribution and apoptosis were detected by flowcytometric analysis.

**Results:**

We discovered that Zfx mRNA was expressed in U251 cells, U87 cells, U373 cells, and A172 cells. The expression level of Zfx is significantly higher in gliomas compared to noncancerous brain tissue. Using a lentivirus-based RNAi approach, Zfx expression was significantly inhibited in human glioblastoma U251 cells. The effects of Zfx knockdown on cell proliferation, cell cycle distribution, and apoptosis were assessed. Inhibition of Zfx expression in U251 cells by RNAi significantly impaired cell proliferation, increased apoptosis, and arrested cells in S phase.

**Conclusions:**

The results of our study demonstrate that the Zfx gene is highly expressed in glioma tissue and in glioma cell lines. Furthermore, Zfx may play a critical role in cell proliferation, cell cycle distribution, and apoptosis of human malignant glioma cells.

## 1. Introduction

Malignant glioma is one of the most common and fatal types of brain tumors in humans [1]. It is the second major cause of cancer-related deaths in both children and young adults, and it is the second fastest growing cause of cancer deaths among those over 65 years old [2-4]. Even when treated with surgery, radiation, and chemotherapy, the median life expectancy of brain cancer patients is only 12-14 months [5,6]. Despite significant gains in the understanding of glioblastoma biology, the prognosis of the disease has not significantly changed over the last 20 years.

The Zfx gene is located on the mammalian X chromosome, at Xp22.12, approximately 23 Mb proximal to this boundary. Zfx is a zinc finger transcription factor that is highly conserved among vertebrates. It contains an acidic transcriptional activation domain, a nuclear localization sequence, and a DNA binding domain consisting of 13 C2H2-type zinc fingers [7]. Zinc finger proteins are characterized by the presence of two cysteines (Cys2) and two histidines (His2) in what is called a zinc finger domain. This domain stabilizes the three-dimensional structure, consisting of a two-stranded antiparallel β-sheet and an α-helix surrounding a central zinc ion [8]. Zinc finger proteins play important roles in multiple biological processes, gene expression, differentiation, and embryonic development [9,10].

To explore the role of Zfx in human malignant glioma, we began with an expression analysis of Zfx mRNA in glioma tumors and glioma cell lines. We also used lentivirus-mediated siRNA targeting of Zfx to down-regulate its expression in the human malignant cell line U251 [11]. Finally, we investigated the effect of Zfx silencing on the cell cycle, apoptosis, and proliferation of U251 cells.

## 2. Materials and methods

### 2.1 Cell line preparation

Human glioma U251 cells, derived from grade IV astrocytomas-glioblastoma multiforme (GBM), and human renal epithelial 293T cells were purchased from Cell Bank Type Culture Collection of Chinese Academy of Sciences (CBTCCCAS, Shanghai, China) and maintained in Dulbecco's modified Eagle's medium (DMEM, GIBCO) with 10% fetal bovine serum (FBS, GIBCO) at 37°C in a humidified atmosphere of 5% CO2.

### 2.2 Clinical sample preparation

Before the study began, written informed consent was obtained from all patients who participated in the study, which was approved by the Ethics Committee of SooChow University. All experiments comply with the current laws of our country. Thirty-five glioma samples were obtained from 35 Chinese patients from March 2009 to Septemper 2010 at the Department of Neurosurgery of The First Affiliated Hospital of Soochow University (Grade I-4cases, Grade II-13cases, Grade III-11cases, and Grade IV-7cases according to the 2007 WHO Classification system). The patients consisted of 19 males and 17 females. The mean ages of the patients at the time of surgery were 38 (male) and 41 (female). All tumors were from patients with newly diagnosed gliomas, who had received no therapy before sample collection. Five adult noncancerous brain tissues were obtained from surgical resections of 5 trauma patients for whom a partial resection of normal brain tissue was required as decompression treatment to reduce increased intracranial pressure under the permission of each patient's family. A portion of these surgically removed samples was immediately snap frozen in liquid nitrogen, and the remaining samples were fixed with formalin and embedded in paraffin for histological studies.

### 2.3 The expression of Zfx in U251 cells, U87 cells, U373 cells, and A172 cells by semi-quantitative RT-PCR

Total RNA from the 4 cell lines was extracted using Trizol reagent (Invitrogen, Inc.) according to the manufacturer's instructions. Briefly, 2 μg of total RNA from each sample was reverse transcribed to single-stranded cDNA. 1 μl of cDNA was used as template for the following PCR.

Zfx-primer:5'-GGCAGTCCACAGCAAGAAC-3'and5'-TTGGTATCCGAGAAAGTCAGAAG-3' product size 237 bp.

Gapdh-primer:5'-GGCAGTCCACAGCAAGAAC-3'and5'-CACCCTGTTGCTGTAGCCAAA-3' product size 121 bp.

The semi-quantitative RT-PCR comprised an initial denaturation at 95°C for 15s, then 22 cycles at 95°C for 5s and 60°C for 30s. PCR products were run on a 2% agarose gel.

### 2.4 The expression of Zfx in 35 pathologically confirmed glioma samples and 5 noncancerous brain tissue samples by real-time quantitative PCR

Total RNA was isolated from glioma tissue using Trizol reagent (Invitrogen USA). cDNA was prepared from 2-6 μg of total RNA using superscript II reverse transcriptase (Invitrogen USA) and random hexamer primers. 1 uL of the cDNA was used for real-time PCR, which was performed to detect Zfx using SYBR Green Mixture (TaKaRa, Japan) according to the manufacturer's protocol. Sequences of both Zfx and GAPDH primers have been previously listed. Real-time PCR comprised an initial denaturation at 95°C for 15s, then 45 cycles at 95°C for 5s and 60°C for 30s. The data were analyzed using GraphPad PRISM4.0 Software. Results were presented as CT values, defined as the threshold PCR cycle number at which an amplified product was first detected. The average CT was calculated for both Zfx and GAPDH, and ΔCT was determined as the mean of the triplicate CT values for Zfx minus the mean of the triplicate CT values for GAPDH. The 2^-ΔΔCT ^method was used to analyze the relative changes in gene expression.

### 2.5 Lentivirus vectors for Zfx small interfering RNA

pGCL-GFP-Lentivirus was used to express small interfering RNAs (siRNAs) targeting the Zfx ORF sequence (Genbank no. NM_003410) (Zfx-siRNA lentivirus). A non-targeting sequence was used as a lentivirus negative control (NC) and was purchased from Shanghai Genechem, Co. Ltd. The template of the experiment:5'-GCCTGAGAATGATCATGGA-3'.

The sequences were cloned into the pGCSIL-GFP (GeneChem, Shanghai, China) to generate the lentiviral vectors. Human renal epithelial 293T cells were infected with Zfx-siRNA lentivirus and NC lentivirus. The interference efficiency of the template was detected by Western blot analysis.

### 2.6 Western blot analysis

Cells were harvested in RIPA buffer that was supplemented with protease and phosphatase inhibitor cocktails. Proteins were separated by SDS-PAGE, transferred onto PVDF membranes, and stained for the following proteins: anti-Zfx (Sigma,1:3000), anti-GAPDH (Santa-Cruz,1:5000). Secondary antibodies conjugated to horseradish peroxidase and ECL Western blotting reagents were used for detection.

### 2.7 U251 cells were infected with Zfx-siRNA lentivirus

Human glioma U251 cells were infected with Zfx-siRNA lentivirus and NC lentivirus. Nontransfected cells were also included as a control. After 3 days of infection, GFP expression was observed by fluorescent microscopy. After 5 days of infection, cells were harvested to determine knock-down efficiency by real-time quantitative PCR.

### 2.8 Cell growth assay

Cell growth was measured via multiparametric high-content screening (HCS). Briefly, human glioma U251 cells at 10 days after being infected with either NC lentivirus or Zfx siRNA lentivirus were seeded at 2000 cells per well in 96-well plates, then incubated at 37°C with 5% CO_2 _for 5 days. Plates were processed with the ArrayScan™ HCS software (Cellomics Inc.) and kept at +4°C for up to 24 h before each day's analysis. The system is a computerized, automated fluorescence-imaging microscope that automatically identifies stained cells and reports the intensity and distribution of fluorescence in each individual cell. Images were acquired for each fluorescence channel, using suitable filters and 20 × objective. In each well, at least 800 cells were analyzed. Images and data were stored in a Microsoft SQL database for easy retrieval.

### 2.9 BrdU incorporation assay

DNA synthesis in proliferating cells was determined by BrdU incorporation. Cells were spread onto 96-well plates and incubated for 24 or 48 hours. 10 uL 1 × 5-bromodeoxyuridine (BrdU) reagent was added from 2 hr to 24 hr, 100 uL Fixing Solution was added to the cells for 30 min. The cells were washed with Wash Buffer and incubated for 60 min with 50 μl 1 × BrdU antibody. After adding 50 μl 1 × Goat anti-Mouse IgG, 50 μl TMB substrate solution was added. Following 30 min incubation, the stop solution was added. The OD was measured at 450 nm using a plate reader.

### 2.10 Flowcytometric analysis of cell cycle distribution

The cells infected with Zfx -siRNA lentivirus or NC lentivirus on the tenth day were plated onto six-well plates in triplicate and incubated at 37°C for 5 days. Cells were then collected, washed twice with ice-cold phosphate-buffered saline (PBS), fixed with 70% ice-cold ethanol, and stained with propidium iodide (PI, 50 μg/ml) in the presence of RNase (100 μg/ml). 1 × 10^4 ^cells were analyzed for the cell cycle phase by flow cytometry.

### 2.11 Detection of apoptosis by flow cytometry

Cell apoptosis was assayed by staining with Annexin V-APC and detected by flowcytometry. For analysis of apoptosis, the cells were stained with 100 ul binding buffer containing 5 ul Annexin V-APC at room temperature in the dark for 10-15 min. Cells were analyzed using flow cytometry. All experiments were performed in triplicate.

### 2.12 Statistical analysis

One-way ANOVA and Student's t-test were used for raw data analysis. Statistical analysis was performed using the SPSS12.0 software package. All values in the text and figures are expressed as the mean ± SD of these observations. A value of P < 0.05 was considered to be statistically significant.

## 3. Results

### 3.1 Measurement of Zfx mRNA in U251 cells, U87 cells, U373 cells, and A172 cells

**Figure 1:**
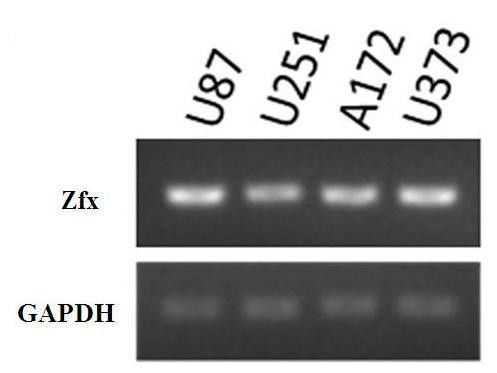
**The expression of Zfx mRNA in the four glioma cell lines was measured by Semi-quantitative RT-PCR**. The symbols are: U251-U251 cells, U87-U87 cells, U373-U373 cells, and A172-A172 cells. A constitutively expressed Gapdh gene was used as an internal control.

We detected the expression of Zfx mRNA in glioma cell lines U251, U87, U373, and A172 by semi-quantitative RT-PCR. Zfx mRNA was expressed in all four cell lines (Figure 1).

### 3.2 The relative expression levels of Zfx mRNA in glioma tissue samples and noncancerous brain tissue samples

**Figure 2:**
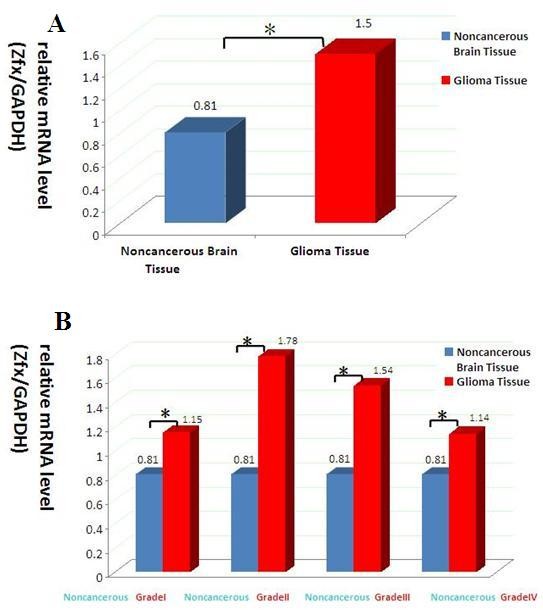
**The expression level of Zfx mRNA in the glioma samples and the noncancerous brain tissue detected by real-time quantitative PCR**. (a) The higher expression level of Zfx in all glioma samples (including the Grade I to Grade IV) versus the noncancerous brain tissue. (p = 0.01). (b) The expression level of each grade glioma versus the noncancerous brain tissue. *P < 0.05.

In order to examine whether there is a significant difference in the expression of Zfx mRNA in glioma tissue compared to noncancerous brain tissue, we performed real-time quantitative PCR. Zfx mRNA is elevated in gliomas compared to noncancerous brain tissue (Figure 2A). We identified correlation between glioma malignancy and Zfx mRNA expression. However, this was not the case for Grade III and Grade IV (Figure 2B).

### 3.3 The interference efficiency of the template was detected by Western blot analysis

**Figure 3:**
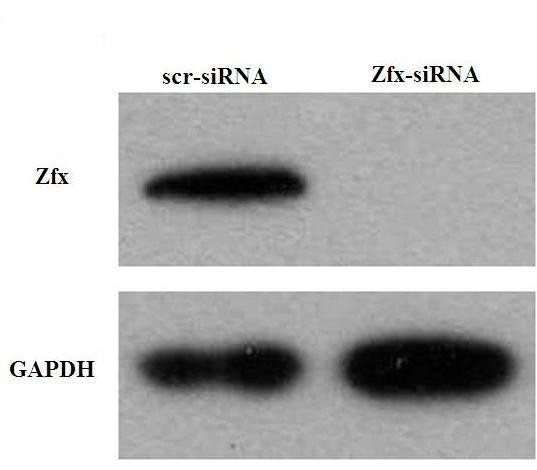
**Protein of Zfx in 293T cells measured by western blot**. Compared with NC, the level of Zfx protein in 293T cells decreased markedly after Zfx expression was silenced by RNAi. Gapdh is a control.

293T cells were infected with Zfx-siRNA lentivirus or NC lentivirus. As shown in Figure 3, Zfx protein level detected by Western blot was greatly reduced in Zfx-siRNA infected cultures, indicating effective knockdown of the target sequence.

### 3.4 Lentivirus-mediated knock-down of Zfx in the human malignant cell line U251

**Figure 4:**
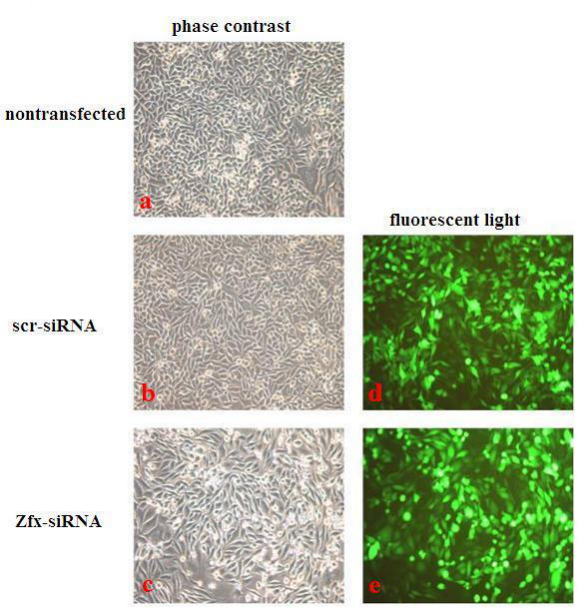
**Human malignant cell line U251 infected with Zfx-siRNA or NC lentivirus and nontransfected cells were examined by fluorescent microscopy and light microscopy at the 3^rd ^day after infection. More than 80% of U251 cells expressed GFP**. There was no significant difference between the negative control group and the nontransfected group, indicating the transfection process has no effect on cells growth. a: 200 × B; b: NC 200 × B; c: NC 200 × B; d: KD 200 × G; e: KD 200 × G. Representative images of the cultures are shown.

**Figure 5:**
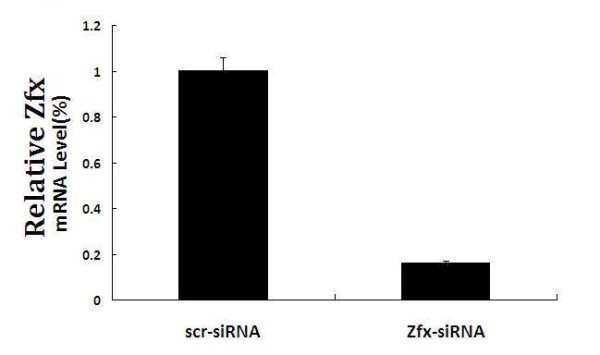
**The cells were lysed and RNAs were extracted to examine Zfx expression levels in U251 cells at the 5^th ^day after infection with Zfx-siRNA lentivirus and NC lentivirus by real-time PCR analysis**. The Zfx mRNA level decreased significantly after zfx knockdown.

To begin to explore the role of Zfx, we knocked down Zfx levels in the human malignant cell line U251. As shown in Figure 4, by 3 days after infection, efficiencies were greater than 80% for both Zfx-siRNA lentivirus and NC lentivirus. There was no significant difference between the negative control cells and the nontransfected cells, indicating that the transfection process itself had no effect on cell growth. Zfx mRNA levels in U251 cells at 5 days after infection with Zfx-siRNA lentivirus and NC lentivirus were assessed by real-time PCR. Zfx-siRNA lentivirus infected cultures had significantly lower levels of Zfx mRNA compared to levels in cultures infected with NC lentivirus (Table 1 and Figure 5).

**Table 1:** CT values of GAPDH and Zfx detected by real-time quantitative PCR

Sample	GAPDH CT valve average	Zfx CT value average	2^-△△CT ^average
scr-siRNA	16.34 ± 0.06	25.89 ± 0.04	1.00 ± 0.06
Zfx-siRNA	16.1 ± 0.02	28.27 ± 0.10	0.16 ± 0.001

**Table 1**:CT values of GAPDH and Zfx detected by real-time quantitative PCR. The Zfx mRNA expression levels in U251 cells at the 5^th ^day after infection with Zfx-siRNA lentivirus and NC lentivirus were analyzed by 2^-△△CT ^method. (P = 0.001).

### 3.5 Knocking down Zfx in human malignant cell line U251 slows cell growth

**Figure 6:**
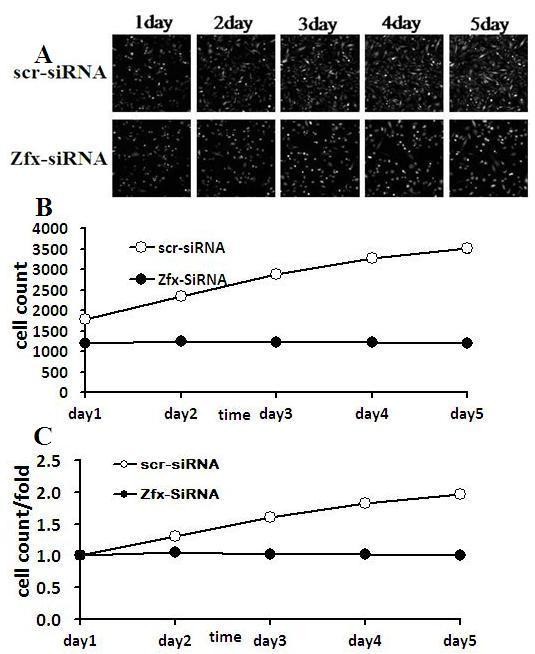
**Effect of down-regulated Zfx on human malignant cell line U251 growth. (A) High content cell imaging assays were applied to acquire raw images (unprocessed by software algorithm) of cell growth**. (B) Human malignant cell line U251 expressing Zfx-siRNA lentivirus and NC lentivirus were seeded in 96-well plates and cell growth was assayed every day for 5 days. (NC vs Zfx -siRNA, P < 0.05). (C) Cell growth rate was monitored on the 2^nd^, 3^rd^, 4^th ^and 5^th ^days by assay. (NC vs Zfx -siRNA, P < 0.05).

**Figure 7:**
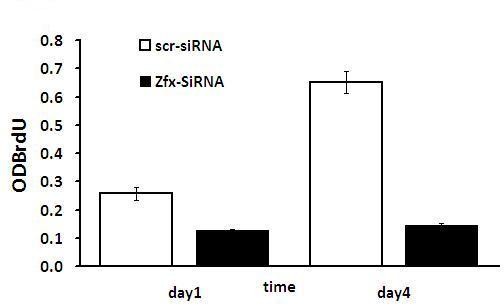
**Down-regulated Zfx in human malignant cell line U251 displayed changes in DNA synthesis**. The DNA synthesis rate was analyzed by BrdU incorporation assay on the 1^st ^and 4^th ^days. (NC vs Zfx -siRNA, P < 0.05).

To explore the function of Zfx on cell growth, U251 cells expressing either Zfx -siRNA lentivirus or NC lentivirus were monitored by high-content screening (HCS) and BrdU incorporation. As shown in Figure 6A, down-regulation of Zfx decreased the total number of cells. U251cells expressing Zfx-siRNA lentivirus and NC lentivirus were seeded in 96-well plates, and cell growth was assayed every day for 5 days (Table 2 and Figure 6B). Cell growth rate was defined as: cell count of Nth day/cell count of 1^st ^day, where n = 2,3,4,5 (Table 3 and Figure 6C). The amounts of DNA synthesized also decreased on the 1^st ^and 4th day after infection with Zfx -siRNA lentivirus (Table 4 and Figure 7). The results of the study show that cell proliferation was significantly inhibited over the course of 4 days. Data shown are the mean results ± SD of a representative experiment performed in triplicate (*n *= 3, indicates *P *< 0.05). These results indicate that knockdown of Zfx expression significantly inhibited proliferation and DNA synthesis of human malignant cell line U251.

**Table 2:** Cell numbers counted by cellomics

AV/num	scr-siRNA	Zfx-SiRNA
day 1	1785.2 ± 86.31	1198.8 ± 53.93
day 2	2337.0 ± 102.75	1254.6 ± 78.84
day 3	2872.0 ± 78.25	1225.4 ± 59.87
day 4	3260.4 ± 75.77	1219.6 ± 56.05
day 5	3505.0 ± 126.04	1198.2 ± 71.14

**Table 2**:Cell numbers counted for 5 days after transfected with Zfx-siRNA lentivirus and NC lentivirus. (The p value = 0.0079. P < 0.05).

**Table 3:** Cell growth rate counted by cellomics

AV/fold	scr-siRNA	Zfx-SiRNA
day 1	1.00 ± 0.00	1.00 ± 0.00
day 2	1.31 ± 0.01	1.05 ± 0.02
day 3	1.61 ± 0.05	1.02 ± 0.02
day 4	1.83 ± 0.07	1.02 ± 0.01
day 5	1.96 ± 0.04	1.00 ± 0.02

**Table 3**: Cell growth rate for the 2^nd^, 3^rd^, 4^th ^and 5^th ^day after transfection with Zfx-siRNA lentivirus and NC lentivirus.

**Table 4:** The amounts of DNA synthesized detected by BrdU incorporation assay

ODBrdu	day 1	day 4
scr-siRNA	0.257 ± 0.024	0.651 ± 0.039
Zfx-siRNA	0.126 ± 0.006	0.146 ± 0.005
p value	0.0082	0.0017

**Table 4**: The amount of DNA synthesized was analyzed by BrdU incorporation on the 4^th ^day and 1^st ^day. (NC vs Zfx -siRNA,P < 0.05).

### 3.6 Knocking down of Zfx in human malignant cell line U251 arrests the cell cycle in S phase

**Figure 8:**
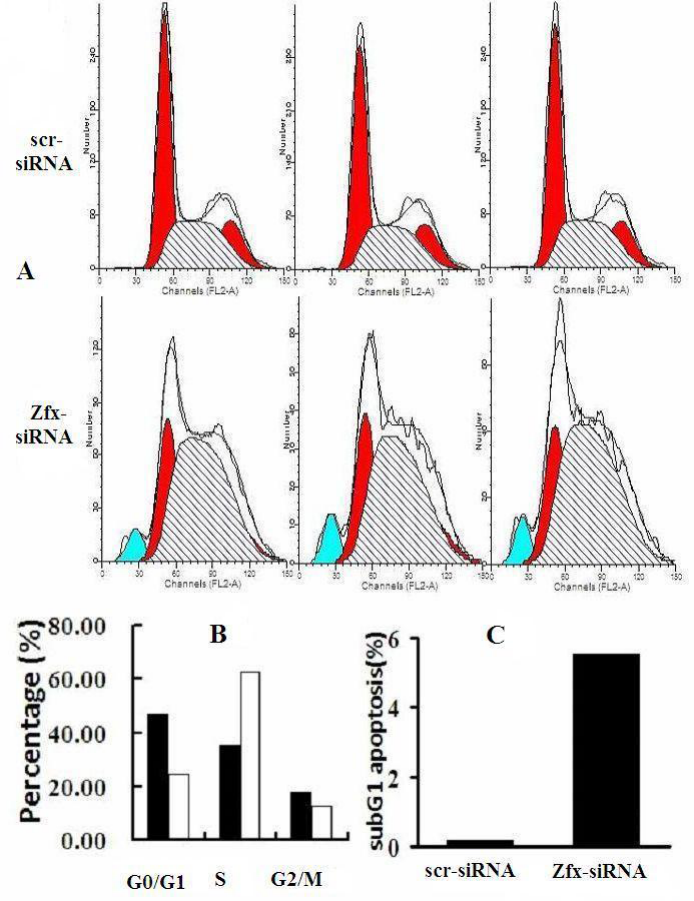
**Knocking down Zfx in human malignant cell line U251 arrested the cell cycle. Knockdown of Zfx expression induced S arrest in U251 cells**. (A) Cell cycle of U251 cells was analyzed by flow cytometry. (B) S cell cycle phase determined by flow cytometry. Compared with NC, Zfx-siRNA cultures showed a significant increase in cells in S (P = 0.003; P < 0.05), compared with NC. (C) Percentage of apoptosis was plotted against U251 cell line. There was a greater amount of apoptosis in the Zfx down-regulated group of human brain glioma U251 cells (P = 0.0009, P < 0.05). The assay showed a marked induction of apoptosis with 5.51% apoptotic for NC group.

To determine whether Zfx is necessary for cell cycle progression of the human malignant cell line U251, we assessed the cell cycle phases in U251 cells by flow cytometry (Figure 8A). The NC Group displayed the following distribution: (G0/G1 46.95%, S 35.12%, G2/M 17.93%), and the Zfx-siRNA Group displayed the following: (G1 24.57%, S 62.82%, G2/M 12.61%). As shown in Figure 8B, compared to control cultures, Zfx -siRNA lentivirus cultures displayed a significant increase in the percentage of cells in S phase (NC 35.12 ± 1.26% vs Zfx -siRNA 62.82 ± 3.696%, *P *= 0.003). A significant increase of cells in the subG1 fraction was observed in the Zfx -siRNA Group compared to the NC Group (NC 0.15 ± 0.046% vs Zfx -siRNA 5.51 ± 0.90%, *P *= 0.0009). (Figure 8C) Taken together, these data suggest that Zfx regulates cell growth and blocks cell cycle progression.

### 3.7 Knocking down of Zfx in human brain glioma U251 cells increase cell apoptosis

**Figure 9:**
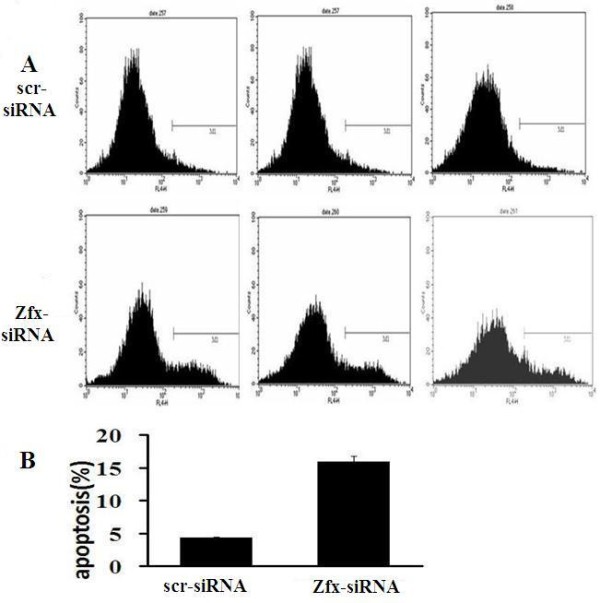
**Knock down of Zfx in human malignant cell line U251 increased cell apoptosis**. (A) Cell death was determined by Annexin V staining and flow cytometry. (B) Zfx-siRNA cultures showed a significant increase in apoptosis compared with NC (P = 0.001; P < 0.05).

To test whether Zfx expression affects human brain glioma U251 cell apoptosis, we knocked down Zfx in this cell line. Cell apoptosis was determined by AnnexinV staining and followed by flow cytometry (Figure 9A). As shown in Figure 9B, cell apoptosis was significantly increased in the Zfx-siRNA Group compared to the NC Group (NC 4.32 ± 0.14% vs. Zfx -siRNA 15.93 ± 0.77%, *P *= 0.001). These results indicate that Zfx expression is a determinant of human brain glioma U251 cell apoptosis.

## 4. Discussion

Recent research shows that Zfx is important for tumorigenesis. Zfx plays a pivotal role in embryonic stem cells and in hematopoietic stem cells. A recent study by Galan Caridad and his colleagues [12] showed that Zfx, is a shared transcriptional regulator of ESC and HSC, suggesting a common genetic basis of self-renewal in embryonic and adult stem cells. Previous work by Gang Hu et al[13] based on a genome-wide siRNA screen in mouse embryonic stem cells found 148 genes whose down-regulation caused differentiation. The study further discovered that a unique module in the self-renewal transcription network is formed by Cnot3, Trim28, c-Myc, and Zfx. The transcriptional targets of this module are enriched for genes involved in cell cycle, cell death, and cancer, and may represent novel anti-cancer targets. Recently, Arenzana et al also reported that Zfx is a novel transcriptional regulator of the B-cell lineage, and one of the common genetic control genes of both stem cell maintenance and lymphocyte homeostasis [14]. The present study discovered that Zfx expression is significantly higher in both Follicular Lymphoma (FL) and Diffuse large B cell lymphoma (DLBCL) and may be used for prognostic purposes in the clinic [15]. Huang D [16] and others found that stem cell-related genes (including OCT-4, SOX-2, BMI-1, and ZFX) were upregulated in SP(side population) cells of human esophageal carcinoma 9706 cells compared with non-SP cells.

To date, most research has focused on the expression and function of Zfx in embryonic stem cells and hematopoietic stem cells. In oncology researches, studies discovered that Zfx is abnormally expressed in prostate cancer, breast cancer, and leukemia [15]. However, its expression and function in human glioma had not been studied. Thus, we first explored the expression levels of Zfx mRNA in four glioma cell lines and found that it was expressed in all of them. We then detected the expression level of Zfx mRNA in glioma samples and in noncancerous brain tissue. Zfx was more highly expressed in glioma samples than in noncancerous brain tissue To some extent, we also found that Zfx expression increased with increasing tumor grade (however, this was not true for Grades III or IV). This may be due to the fact that Zfx mutations may occur at high frequency in high grade malignant gliomas. In order to assess Zfx function in glioma cell lines, we infected human glioblastoma U251 cells with Zfx-siRNA lentivirus or control lentivirus. Compared to controls, Zfx-siRNA treated cells showed decreased proliferation, increased apoptosis, and an increase in the proportion of cells in S and subG1 phases. Thus, Zfx promotes U251 cell growth.

Our data suggest that Zfx may be related to cell cycle checkpoints in U251 cells. The cell has developed a series of checkpoints to ensure quality control over proliferation. In particular, S phase represents a critical period for cells to commit to proliferation or undergo growth arrest [17]. Understanding the regulation of the S phase transition is central to the study of many diseases, particularly cancer [18,19]. The cell cycle is a well regulated process that depends on the combined action of both cell cycle activators and inhibitors [20].

With the emergence of the cancer stem cell theory, many researchers now believe that glioma stem cells are at the root of disease recurrence due in large part to their natural drug resistance and insensitivity to radiation therapy, Thus, successful tumor treatment likely depends on complete eradication of tumor stem cells [21]. Cancer stem cells with self-renewal capability can constitute a tumor by proliferation and differentiation, key processes in the formation, proliferation, and invasiveness of cancer [22,23]. Zfx may be a key gene involved in the molecular basis of stem cells, and this also potentially implicates it in cancer stem cell biology. However, whether Zfx plays a role in glioma stem cell self-renewal growth is currently unknown.

In summary, our study highlights critical roles for Zfx in the human malignant glioma cell line U251. This study may provide the basis for further exploration of the role of Zfx in the occurrence and development of human glioma. We will continue to work on the mechanism by which Zfx influences glioma cell biology.

## Competing interests

The authors declare that they have no competing interests.

## Authors' contributions

YZ conceived of the study, and participated in its design and coordination and helped to draft the manuscript. TS carried out the molecular genetic studies. YH participated in its design and coordination. ZS participated in the conception and the design of the analysis. All authors read and approved the final manuscript.
